# Role of SPRY4 in health and disease

**DOI:** 10.3389/fonc.2024.1376873

**Published:** 2024-04-15

**Authors:** Hao Pan, Renjie Xu, Yong Zhang

**Affiliations:** Department of Hepatobiliary Surgery, Union Hospital, Tongji Medical College, Huazhong University of Science and Technology, Wuhan, China

**Keywords:** SPRY4, cancer, development, proliferation, metastasis, tumor suppressor, apoptosis, oxidative stress

## Abstract

SPRY4 is a protein encoding gene that belongs to the Spry family. It inhibits the mitogen-activated protein kinase (MAPK) signaling pathway and plays a role in various biological functions under normal and pathological conditions. The SPRY4 protein has a specific structure and interacts with other molecules to regulate cellular behavior. It serves as a negative feedback inhibitor of the receptor protein tyrosine kinases (RTK) signaling pathway and interferes with cell proliferation and migration. SPRY4 also influences inflammation, oxidative stress, and cell apoptosis. In different types of tumors, SPRY4 can act as a tumor suppressor or an oncogene. Its dysregulation is associated with the development and progression of various cancers, including colorectal cancer, glioblastoma, hepatocellular carcinoma, perihilar cholangiocarcinoma, gastric cancer, breast cancer, and lung cancer. SPRY4 is also involved in organ development and is associated with ischemic diseases. Further research is ongoing to understand the expression and function of SPRY4 in specific tumor microenvironments and its potential as a therapeutic target.

## 1 Introduction

SPRY4, also known as sprouty RTK signaling antagonist 4(Sprouty4), is a gene that encodes a protein belonging to the Spry family. This family consists of proteins that are rich in cysteine and proline (1, 2). The SPRY4 protein is an inhibitor of the receptor transduction MAPK signaling pathway. It is an intracellular protein that translocates to the plasma membrane upon activation, with its structural domain located in the cytoplasmic membrane (3). With a molecular weight of approximately 32.541 KDa, SPRY4 is involved in various cellular biological functions under both physiological and pathological conditions. In addition to its role in embryonic development and organogenesis (4, 5), SPRY4 is also associated with cell apoptosis and proliferation, oxidative stress, inflammatory response, and ischemic injury (1, 6–11). Furthermore, SPRY4 plays a significant role in the occurrence and development of tumors (12). This review article summarizes recent research on SPRY4, focusing on its research progress in various diseases.

## 2 SPRY4 protein characterization

The discovery of the Drosophila Sprouty (dSpry) protein by Hacohen et al. in 1998 revealed its inhibitory function in the Ras/MAPK signaling pathway. Subsequently, four members of the Spry family were identified in mammals, namely Spry1, Spry2, Spry3, and Spry4 (13). These SPRY proteins contain several highly conserved domains, including the N-terminal c-Cbl binding domain (CBD), the serine-rich motif (SRM), and the C-terminal cysteine-rich domain (CRD) (14). The amino acid sequence similarity within the entire C-terminal domain ranges from 56% to 70% among the four mouse proteins. In the N-terminal domain, the amino acid sequence similarity between murine SPRY4 (mSPRY4) and mSPRY1 or mSPRY2 is only 25%. The main difference between dSpry and mSPRY is observed in the N-terminal domain, while the C-terminal domain is highly conserved with an amino acid sequence similarity of approximately 44% to 52% (15).

**Figure 1:**
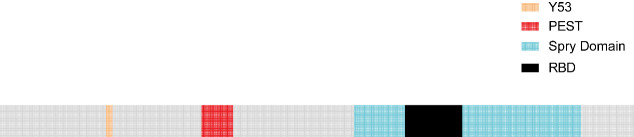
Blue region: C-terminal region rich in cysteine ​​residues, which contains a domain that binds to Raf1 (Raf1 binding domain, RBD, black region);Yellow region: Amino-terminal tyrosine residue; Red region: PEST sequence.

In 2002, Onno C. Leeksma and colleagues discovered a new Spry gene in umbilical artery smooth muscle cells (SMCs), which is a homologous gene of mSpry4 and named human SPRY4 (hSPRY4). The hSPRY4 gene is located on chromosome 5q31.3 and encodes a protein of 322 amino acids (Refer to the **Table 1** for details). It contains a cysteine-rich C-terminal region (CRD), three potential SH3 binding sequences, a PEST sequence, and a tyrosine and serine residue (16, 17). The CRD also contains a highly conserved motif that mediates binding to Raf1 (Raf1-binding domain, RBD) (**Figure 1**).

**Table 1:** Basic information of human and mouse SPRY4 protein.

Gene Symbol	Chromosome Location	Molecular Weight (kDa)	protein isoelectric point
hSPRY4	5q31.3	32.6	8.25
mSPRY4	18 B3	32.6	8.25

**Figure 2:**
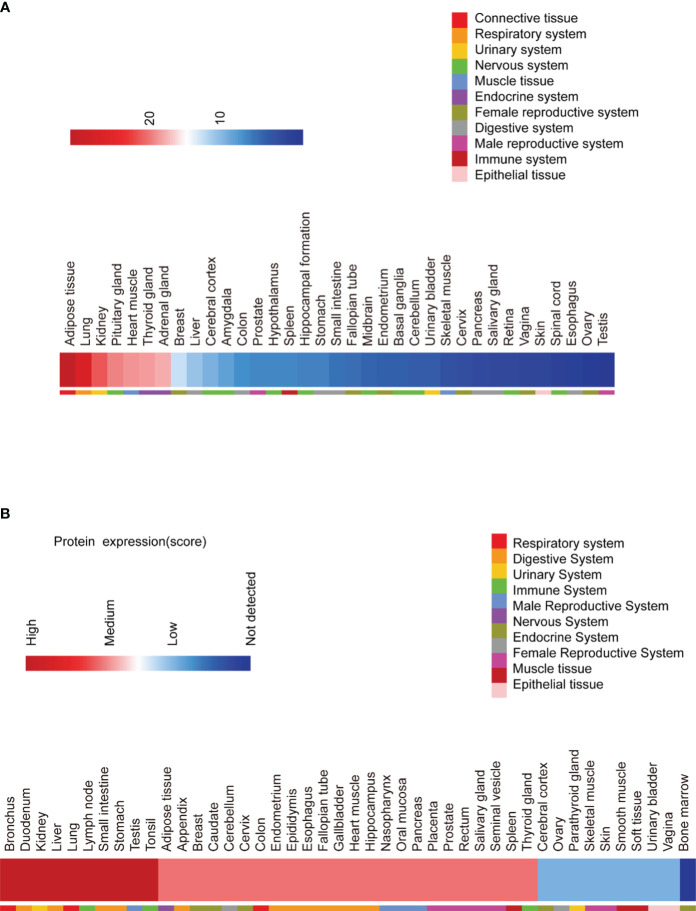
RNA and protein expression of SPRY4 proteins. **(A)** RNA expression of SPRY4 across tissues from The Genotype Tissue Expression (GTEX) Project (https://www.gtexportal.org/home). **(B)** Protein expression of SPRY4 across tissues from The Human Protein Atlas (https://www.proteinatlas.org).

Analysis of RNA expression profiles shows that SPRY4 is widely expressed in human tissues (**Figure 2A**; Genotype-Tissue Expression Project, https://www.gtexportal.org/home). Immunohistochemical staining of different human tissue sections also reveals similar expression patterns (**Figure 2B**; The Human Protein Atlas, https://www.proteinatlas.org). The SPRY4 protein is highly expressed in organs such as the bronchi, lungs, stomach, duodenum, small intestine, and liver. On the other hand, at the RNA level, the top 6 tissues with high expression are adipose tissue, lungs, kidneys, pituitary gland, cardiac muscle, and thyroid (**Figure 2**).

## 3 SPRY4 Function

It is well known that the spatial structure of proteins determines their function. In all SPRY proteins, the carboxy-terminal domain not only allows the protein to translocate to the cell membrane but also plays a role in inhibiting the MAPK pathway (16). The amino acid sequence of hSPRY4 contains three potential SH3 binding domains, which are related to its regulatory function in signal transduction. Additionally, SPRY4 includes a PEST sequence (17). PEST sequences are specific amino acid sequences that typically contain at least one proline (P), one glutamic acid (E) or aspartic acid (D), and one serine (S) or threonine (T), flanked by lysine (K), arginine (R), or histidine (H) residues (17, 18). The removal of the PEST region (19) or mutations in the PEST sequence (20) can lead to increased protein stability. Furthermore, researchers have transferred PEST sequences to stable proteins, observing that the resulting chimeric proteins degrade rapidly (21), which reinforces the functional significance of PEST sequences in protein degradation. Therefore, it is speculated that the PEST region in SPRY4 plays a key role in the degradation of this protein. Furthermore, SPRY4 also regulates Cellular Behavior through interactions with other molecules. Further investigation is needed to comprehensively explore the potential biological functions of SPRY4.

### 3.1 The SPRY4 protein serves as a negative feedback inhibitor of RTK signaling

The MAPK signaling pathway is involved in various cellular physiological activities, such as growth, development, differentiation, and apoptosis, making it a crucial target for tumorigenesis. When the Ras/MAPK signaling pathway is activated by fibroblast growth factor (FGF), epidermal growth factor (EGF), vascular endothelial growth factor (VEGF), platelet derived growth factor (PDGF), nerve growth factor (NGF), and insulin, SPRY4 protein is induced and acts as a negative feedback inhibitor of this signaling pathway. In 2001, researchers in Japan found that overexpression of mSPRY4 protein in 293 cell lines inhibited the activation of extracellular regulating kinase (ERK) induced by FGF, but had no effect on the activation of ERK induced by EGF (22). SPRY4 might possess ligand specificity, which means it could have different effects on signal responses induced by different growth factors. Further studies conducted by researchers have elucidated the mechanism by which SPRY4 inhibits FGF-induced ERK activation. SPRY4 binds to free Son of sevenless 1(SOS1) or the Grb2-Sos1 complex, thereby disrupting the interaction between growth factor receptor-bound protein 2 (Grb2) and Sos1, thereby inhibiting FGF-induced ERK activation. Through its C-terminal domain, SPRY4 forms hetero- and homo-oligomers with other subtypes of SPRY, with the SPRY4-SPRY1 oligomer being more effective in suppressing FGF-induced ERK activation (23).

However, in 2002, Onno C. Leeksma et al. found that hSPRY4 can inhibit the activation of insulin and EGF receptor-mediated MAP kinase by interfering with rat sarcoma (Ras) activation. SPRY4 may impair the formation of active GTP-Ras and exert its inhibitory effects at the level of Ras or its upstream components. The MAP kinase activated by constitute active V12 Ras is not affected by hSPRY4, indicating that the observed inhibition in insulin or EGF stimulation occurs through interference with Ras activation (17). The findings of Sang Hoon Lee et al. once again support this view - SPRY4 uncouples RTK signaling from Ras activation, inhibiting FGF and VEGF signaling transduction (24). Raf is activated through both Ras-dependent and Ras-independent mechanisms. In 2003, Sasaki et al. defined a novel Ras-independent Raf-dependent signaling pathway triggered by VEGF, namely the PLCγ1-PKC-ERK signaling pathway. The binding of SPRY4 to Raf1 is essential for the inhibition of VEGF-induced ERK activation, while the amino-terminal of SPRY4 contains conserved tyrosine residues necessary for inhibiting fibroblast growth factor signaling transduction (25).

In 2009, T Ayada et al. discovered that SPRY4 can be activated not only by various growth factors but also by ligands of G protein-coupled receptors (GPCRs), such as lysophosphatidic acid (LPA) and sphingosine-1-phosphate (S1P). The CR domain of the SPRY4 protein binds to phosphatidylinositol bisphosphate (PIP2), which effectively shields it from phospholipase Cγ2 (PLCγ), thereby inhibiting the hydrolysis of PIP2. This inhibition does not interfere with the activation of PLCγ. As a result, the signaling pathways downstream of protein kinase C (PKC), as well as the mobilization of Ca2+ induced by VEGF-A and LPA, are blocked (26).

**Figure 3:**
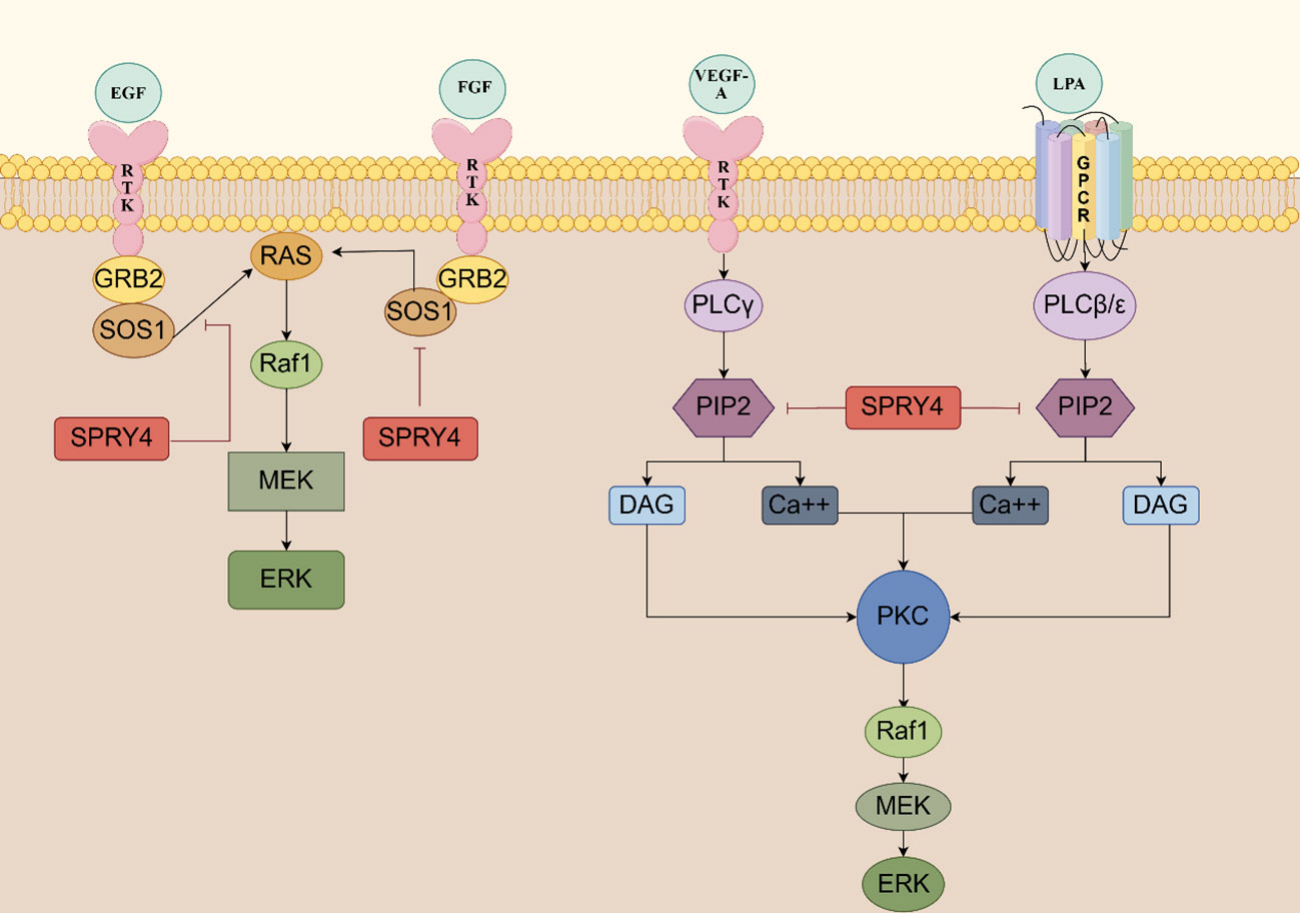
The SPRY4 protein serves as a negative feedback inhibitor of RTK signaling SPRY4 binds to free sos1 or the GRB2-SOS1 complex, thereby disrupting the interaction between GRB2 and SOS1, thereby inhibiting FGF-induced ERK activation. SPRY4 may impair the formation of active GTP-ras and exert its inhibitory effects at the level of Ras or its upstream components. The CR domain of the SPRY4 protein binds to PIP2, which effectively shields it from PLCγ, thereby inhibiting the hydrolysis of PIP2 (https://www.figdraw.com).

The SPRY4 protein inhibits the activation of protein kinase D (PKD) by negatively regulating the S1P/PLC-γ/PKC pathway. Additionally, SPRY4 also suppresses the activation of ERK and AKT induced by S1P (27). These research findings reveal the complex role of SPRY4 in regulating the MAPK signaling pathway. It not only exhibits different responses to various growth factors but also participates in negative regulation of multiple signaling pathways. These discoveries contribute to our understanding of the fine-tuning mechanisms of cellular signaling and may provide important clues for the development of therapeutic strategies targeting the MAPK signaling pathway (**Figure 3**).

### 3.2 SPRY4 interferes with cell proliferation and influences cell fate

SPRY4 overexpression significantly inhibits colon cancer cell proliferation by suppressing human colorectal cancer (CRC) cell viability and impeding cancer cell colony formation, which may be achieved by regulating the EZH2/MDM2/p53 pathway (8). SPRY4 can act as a proliferation inhibitor partly by inducing a significant arrest of the cell cycle in the G1-G0 phase in CRC (28). QiuBo et al. demonstrated by *in vitro* and *in vivo* experiments (xenograft mice) that SPRY4 reduction significantly promoted the growth of human cholangiocarcinoma cells. They further found that SPRY4 promoted cholangiocarcinoma progression by promoting cancer cell proliferation rather than by inhibiting cancer cell apoptosis. These effects may be related to SPRY4 inhibition of FGFR-ERK pathway activation and cell cycle blockade (12). In non-small cell lung cancer, peroxisome proliferator-activated receptor γ (PPARγ), a downstream target of Wnt7A/Fzd9 signaling, inhibits non-small cell lung cancer cell proliferation by up-regulating SPRY4 expression levels through regulating SPRY4 promoter activity (29). Notably, no inhibitory effect of ectopic expression of SPRY4 on proliferation was observed in osteosarcoma-derived cells, possibly because the activation of the MAPK/ERK pathway in osteosarcoma cells is not primarily dependent on the regulation of SPRY4 (30). These results suggest that SPRY4 may have different effects in different tumors. SPRY4 also has the potential to promote proliferation in non-tumor cells. For example, SPRY4 regulates nourishing cell proliferation by modulating the expression and activation of IFN-γ-induced STAT1. Knocking down SPRY4 in HTR8 cells significantly increases the proportion of EdU-labeled cells, promotes the expression of proliferating cell nuclear antigen (PCNA) involved in cell DNA replication, and increases the fluorescence intensity of Ki67, thereby promoting cell proliferation (9). These results indicate that SPRY4 plays a complex role in regulating cell proliferation. It can act as a tumor suppressor, inhibiting the proliferation and migration of cancer cells, but it may also promote cell proliferation in non-tumor cells. This suggests that the function of SPRY4 is multifaceted and may depend on specific cell types and physiological environments. These findings provide potential targets for future cancer treatment and are of significant importance for understanding the molecular mechanisms of cell proliferation and cancer development. When considering therapeutic applications, careful evaluation of the specific role of SPRY4 in different tumor or cellular contexts is necessary.

### 3.3 SPRY4 inhibits cell spread and migration

Cell spread and cell migration are part of the process of rearranging the cell skeleton, and these cell behaviors play important roles in tissue and organ development, regeneration, inflammation, and cancer. SPRY4 exerts its inhibitory effects on cell spread and migration by inhibiting different pathways. The phosphorylation of cofilin by testis protein kinase 1 (TESK1) regulates actin cytoskeleton remodeling and plays a key role in integrin-mediated actin rearrangement and cell spread (31–33). SPRY4 binds to TESK1 through its cysteine-rich domain (CRD) at the C-terminal, negatively regulates its kinase activity, and therefore inhibits integrin-mediated cell spread and migration. The MEK inhibitor PD98059 has no effect on cell spread and migration, suggesting a new cellular function of SPRY4 proposed by Tsumura et al., which is the inhibition of TESK1 activity and integrin-mediated cell spread and migration, independent of the Ras/ERK pathway (34). The discovery by Yan Gong et al. further confirms that SPRY4 inhibits cell migration through a Ras/ERK-independent mechanism, but they found that the use of Src family kinase (SFK) inhibitor PP2 significantly inhibits cell migration. Further studies revealed that SPRY4 partially inhibits endothelial cell migration and adhesion by reducing the phosphorylation of c-Src and integrin beta-3, leading to the inhibition of c-Src activation and the reduction of integrin beta-3 protein levels (35, 36).In *in vitro* experiments, it has been shown that mSPRY4 is an inhibitor of PANC-1 cell migration. mSPRY4 disrupts the subcellular localization of protein tyrosine phosphatase 1B (PTP1B), thereby interfering with integrin signaling and weakening the migration and adhesion of PANC-1 cells. Moreover, SPRY4 can also inhibit cell migration through a Ras/ERK-dependent mechanism (12, 24, 29). In summary, SPRY4 plays an important regulatory role in cell spreading and migration by modulating multiple signaling pathways and protein activities. It inhibits the ability of cells to spread and migrate by regulating cytoskeletal rearrangement processes. These findings provide important clues for further research on cell migration and invasion mechanisms and offer new targets for the development of therapeutic approaches for related diseases.

### 3.4 SPRY4 overexpression promotes inflammation and oxidative stress

Researchers have found that overexpression of macrophage SPRY4 exacerbates sepsis-induced acute lung injury, leading to increased inflammation scores and impaired lung function. It also exacerbates the mortality of septic mice. Furthermore, compared to the control group, mice with macrophage SPRY4 overexpression exhibited higher expression levels of IL-1β and IL-6 in the lung lavage fluid, increased infiltration of macrophages and neutrophils, and increased NF-κB transcriptional activity in lung tissue (1). Similarly, Y Goldshmit et al. found that Spry4 knockout mice showed reduced inflammatory responses in spinal cord injury, including decreased secretion of tumor necrosis factor alpha (TNFα) and decreased invasion of macrophages/neutrophils into the lesion site (10). Spry4-/- mice exhibited decreased proliferation of astrocytes after spinal cord injury, and it has been reported that astrocytes can respond to inflammatory signals and promote inflammation (37). Similarly, Tomohiro Fukaya et al. found that the loss of SPRY4 inhibited the expression of IL-1β receptors and reduced Th17 cell generation in experimental autoimmune encephalomyelitis in mice, thereby improving the condition (6). During sepsis, mice with macrophage SPRY4 overexpression exhibited increased levels of reactive oxygen species (ROS) in lung tissue. The expression levels of nuclear factor erythroid 2-related factor 2 (NRF2) protein and overall antioxidant capacity were decreased (1). However, Sunghyun Park et al. found that overexpression of SPRY4 in healthy chondrocytes led to a decrease in reactive oxygen species (ROS) generation. This contradicts the previous research results (38). NRF2 and NF-κB are key regulatory factors involved in cellular responses to inflammation and oxidative stress. SPRY4 can regulate the expression levels of NRF2 and NF-κB transcription factors, which may explain its involvement in the regulation of inflammation and oxidative stress. These research findings suggest that the expression levels and functions of SPRY4 may vary depending on the cell type, tissue environment, and disease state. In some cases, SPRY4’s function may be associated with pro-inflammatory responses, while in other cases, it may have anti-inflammatory or protective effects. These findings are of significant importance for understanding the role of SPRY4 in inflammation and oxidative stress and may provide potential targets for the treatment of related diseases. However, due to conflicting results among studies, further research is needed to elucidate the specific role of SPRY4 in different biological contexts.

### 3.5 SPRY4 induces cell apoptosis

*In vitro* experiments have shown that SPRY4 overexpression inhibits cell proliferation, impairs DNA synthesis, and accelerates apoptosis in HTR8 cell lines. The expression levels of pro-apoptotic molecules Bax and Cleaved Caspase-3 increase, as well as the proportion of apoptotic cells, while the expression level of anti-apoptotic protein Bcl2 decreases (9). Consistent with previous results, researchers found that overexpression of SPRY4 induces apoptosis in human colorectal cancer cell lines (SW480), increasing the apoptotic rate, upregulating the expression of pro-apoptotic proteins (Bax, Cleaved Caspase-3), and downregulating the expression of anti-apoptotic proteins (Bcl2 and EZH2) (8). It has been reported that silencing EZH2 can reduce apoptosis and inflammatory responses in renal tubular epithelial cells (39). Therefore, it is speculated that SPRY4 may induce apoptosis by inhibiting EZH2. However, contrasting results were observed in QBC939 human cholangiocarcinoma cells, where knocking down SPRY4 did not affect the apoptotic cell rate or the expression levels of apoptotic-related proteins Bax, Caspase-3, and Bcl2 (12). This suggests that SPRY4 does not impact apoptosis in QBC939 cells. Indeed, these results indicate that SPRY4 may have different roles in different cell types and biological contexts. The impact of SPRY4 on apoptosis may be influenced by cellular context and other potential molecular mechanisms that may be present. Therefore, further research is needed to understand in detail the specific mechanisms by which SPRY4 regulates apoptosis in different cell types and its potential therapeutic significance.

## 4 SPRY4 and organ development

Since the discovery of Spry in 1998, a growing body of evidence has supported its critical role in regulating a variety of physiological and pathological processes. SPRY4 is a protein that regulates and influences several organ developmental processes. Specifically, SPRY4 is involved in the development of the limbs, midbrain, head and trunk, teeth, pancreas, blood vessels, bones, kidneys, muscles, lungs, nerve axons, and adipocytes. These findings contribute to our better understanding of the function of SPRY4 during development. We hypothesize that downregulation of SPRY4 expression may promote carcinogenesis by overstimulating the Ras/ERK pathway (Refer to the **Table 2** for details).

**Table 2:** SPRY4 and Organ development.

Investigator	SPRY4	Organ Development
Minowada et al (15)	mSPRY4	Limb Development
Furthauer et al (40)	zSpry4	Midbrain Development
Zhang et al (41)	mSPRY4	Craniofacial and trunk development
Price et al (42)	hSPRY4	Kidney Development
Laziz et al (43)	hSPRY4	Muscle regeneration
Klein et al (44)	mSPRY4	Rodent incisor growth and development
Jaggi et al (45)	mSPRY4	Pancreatic development
S H Lee et al (24)、 Yan Gong (35)	mSPRY4	Angiogenesis
Lijie Tian et al (46)	mSPRY4	Osteogenic and lipogenic differentiation
Wei Ding et al (4) Anne-Karina T Perl et al (47)	hSPRY4	Lung development
Ferrero Restelli, F et al (48) Hausott B et al (49)	mSPRY4	Neuronal axonal growth

mSPRY4, mouse SPRY4, hSPRY4, human SPRY4, zSpry4, zebrafish Spry4.

## 5 The role of SPRY4 in malignant tumors

SPRY4 participates in the occurrence and development of tumors by regulating cellular signaling pathways. The regulatory role of SPRY4 in tumor development is mainly achieved through the following aspects: ①Inhibition of cell proliferation: SPRY4 inhibits tumor cell proliferation by regulating cell proliferation-related signaling pathways, such as the Ras-MAPK and PI3K-Akt pathways (12, 50, 51). ②Cell differentiation: SPRY4 plays an important role in normal cell differentiation processes (46, 52, 53). Additionally, SPRY4 inhibits tumor development by regulating cell differentiation in rhabdomyosarcoma and non-small cell lung cancer (29, 54). ③Inhibition of invasion and metastasis: SPRY4 also plays a significant role in regulating tumor cell invasion. It can inhibit tumor cell invasion and metastasis by modulating key factors such as cell adhesion, cytoskeleton, and extracellular matrix degradation. SPRY4 can suppress the activity of matrix metalloproteinase 9, increase the expression of TIMP1 and CD82, and inhibit tumor cell invasion and metastasis (29). SPRY4 induces macrophage-induced protrusion formation and cytoskeletal changes in undifferentiated thyroid cancer cells, thereby increasing cancer cell invasiveness (55). SPRY4 overexpression induces remodeling of the actin cytoskeleton and inhibits extracellular matrix proteolysis, thus inhibiting invasion of breast ductal carcinoma cells (56). ④Regulation of tumor microenvironment: SPRY4 may regulate tumor occurrence by influencing cell-cell interactions and the release of signaling molecules in the tumor microenvironment. SPRY4 may act as a mediator of communication between macrophages and undifferentiated thyroid cancer cells, exerting tumor-suppressive effects (55). Moreover, as a tumor suppressor, SPRY4 can inhibit angiogenesis and increase vascular permeability in Lewis lung cancer in mice (27).

When the function of SPRY4 is affected by gene mutations, excessive methylation, epigenetic modifications, and mRNA and protein stability, its ability to regulate cellular signaling pathways may be impaired. This can contribute to the occurrence and development of tumors.①Gene mutations: Missense mutations in the coding sequence of the SPRY4 gene generate SPRY4 protein variants (amino acid residue 241 changes from tyrosine to serine). Mutations in the SPRY4 protein inhibit cell migration in osteosarcoma-derived cell lines (57). Another SPRY4 protein variant is generated when the cytosine at the 701st nucleotide position in the SPRY4 coding sequence mutates to thymine, resulting in threonine-to-methionine substitution at amino acid residue 234. This variant promotes the proliferation of thyroid cancer cells (58). ②mRNA and protein stability: In non-small cell lung cancer, significantly upregulated KSRP protein promotes rapid decay of SPRY4 mRNA, leading to increased cell proliferation, migration, and invasion, thereby promoting lung cancer development (59). Additionally, several microRNAs, such as miR-411-5p (rhabdomyosarcoma), miR-411 (non-small cell lung cancer), miR-18a (non-small cell lung cancer), miR-92a (non-small cell lung cancer), miR-1908 (glioma), and miR-181 (breast cancer), downregulate SPRY4 by directly targeting and degrading SPRY4 transcripts in various cancer cells, promoting tumor occurrence and development (54, 60–63). However, miR-302s act as an oncogene in TGCT by inducing SPRY4 expression, activating the MAPK/ERK pathway, and inhibiting apoptosis through increased survivin expression (50). In normal monkey kidney cells, E3 ubiquitin ligase SIAH2 leads to a decrease in SPRY4 protein levels, but its effect is relatively minor under the influence of SIAH2 (64). ③Epigenetic modifications: The expression levels of SPRY4 may be significantly downregulated in certain tumors, possibly due to changes in gene epigenetic modifications. In hepatocellular carcinoma-resistant patients, histone deacetylase 4 (HDAC4) modifies the chromatin configuration within the SPRY4 promoter region, leading to transcriptional inhibition of the SPRY4 gene (65). CCAT1-mediated histone methylation (H3K9me2 and H3K9me3) may also contribute to decreased expression of SPRY4 in esophageal squamous cell carcinoma, promoting cell growth and migration (66). ④Excessive methylation: Excessive methylation of the SPRY4 gene promoter region can lead to gene silencing and downregulation of expression, resulting in the loss of its regulatory role in cellular signaling transduction. High methylation in the SPRY4 promoter region has been observed in patients with prostate cancer (67), colorectal cancer (68), and familial testicular cancer (69), leading to transcriptional inactivation of *SPRY4* and promoting tumor occurrence and development. In human colorectal cancer tumors, overexpression of UHRF1 upregulates SPRY4 transcriptional activity by regulating 5-hydroxymethylcytosine levels in the SPRY4 locus, promoting tumor development (70).

### 5.1 Colorectal cancer

Colorectal cancer (CRC) is a common malignant tumor in the digestive tract, and its metastasis is mainly related to uncontrolled proliferation. In 2021, Jia Guo et al. discovered that the expression level of SPRY4 in NCM460 cell lines, among four human CRC cell lines (SW620, SW480, LOVO, and HCT116), was the highest, while SW480 cell line had the lowest expression level. As a tumor suppressor gene, SPRY4 inhibits the proliferation, migration, and invasion of SW480 cells by regulating the MDM2/p53 pathway mediated by EZH2, and promotes apoptosis. SPRY4 overexpression inhibits tumor formation *in vivo* by reducing tumor size and weight (8). In 2023, Alexei J. Stuckel et al. analyzed the sequencing data of SPRY4 in gastric cancer tissues from the GEO database and TCGA database and found that the transcript levels of SPRY4 were increased in colorectal cancer patients compared to adjacent colonic and healthy mucosal control groups. This may be related to hypomethylation in the distal promoter region of CRC patients (68). DNA methylation is closely related to cancer development (71), and DNA methylation changes include hypermethylation and hypomethylation. Generally, high DNA methylation in the promoter region of a gene indicates gene silencing, while low DNA methylation indicates gene activation (72). Interestingly, Zhou et al. reported high methylation in the promoter region of SPRY4 in a limited number of CRC patients and found that the expression of SPRY4 was decreased in colorectal cancer tissues, significantly correlated with tumor invasion and advanced TNM stage. Furthermore, low expression of SPRY4 predicted poor prognosis in colorectal cancer (28). These research findings suggest that SPRY4, as a tumor suppressor in colorectal cancer, may have a complex role and be regulated by multiple factors, including gene expression regulation and epigenetic modifications.

(Refer to the **Table 3** for details).

**Table 3:** The role of SPRY4 in Diseases.

Disease	Species and tissue or cell type	Stress Condition	Alteration of SPRY4 expression	Method for SPRY4 detection	Biological function	Ref
Pancreatic β-cell cancer	Rip1Tag2; Rip1rtTA; tet(O)7mSpry4 mouse PANC-1	Doxycycline; Nude Miceβ Tumor Cell mode; SPRY4 overexpression cell mode	Down-regulation	WB IHC IF	Inhibits cell Migration and Adhesion, but not Affects β-Cell Carcinogenesis and Progression;	Jaggi et al (45)
Human Prostate Cancer	Human prostate tissue;Human prostate cancer cell lines:PC3, DU145, LNCaP; HUVEC	SPRY4 overexpression cell mode	Down-regulation	ISH RT-qPCR	Inhibit cell migration	Jianghua Wang et al (67)
Non-small cell lung cancer	NSCLC cell lines: H157, H2122 Human Normal Lung Epithelial Cell:Beas2B; human bronchial epithelial cell:HBEC	SPRY4 knockdown and overexpression cell mode	Down-regulation	RT-qPCR	Inhibit cell growth, migration, invasion, and epithelial-mesenchymal transition	Meredith A Tennis et al (29)
Non-small cell lung cancer	Human lung tissue; HBEC A549 SPC-A1 H1299 PC-9 95-D	SPRY4 depletion and overexpression cell mode	Down-regulation	RT-qPCR GEO database	Inhibit cell proliferation and migration	Caiyan Zhang et al (60)
Hepatocellular carcinoma	Human liver tissue	N/A	Down-regulation	RT-qPCR	As tumor suppressor	Sirivatanauksorn et al (73)
Colorectal cancer	Human colon tissues; Human colorectal cancer cell lines:HCT-116, Lovo	5-azacytidine; SPRY4 overexpresssion cell mode; HCT-116 cell-line-derived subcutaneous tumor mode	Down-regulation	RT-qPCR	Inhibit colorectal cancer cell proliferation; Associated with favorable prognosis	Zhou et al (28)
Epithelial ovarian cancer	Human ovary	N/A	Down-regulation	IHC	N/A	Masoumi-Moghaddam S et al (74)
Testicular germ cell tumour	Human testicular tissue; NT2-D1, 833 K	SPRY4 depletion cell mode	Up-regulation	RT-qPCR WB	Knockdown of SPRY4 resulted in reduced cell growth, migration and invasion.	Mrinal K Das et al (51)
Acute myeloid leukemia	Human bone marrow tissue	N/A	N/A	N/A	Higher levels of SPRY4 expression are associated with better prognosis	Sabine Kayser et al (75)
Epithelial ovarian cancer	Human EOC cell lines :BG-1, CaOV3, OVCAR3 and SKOV3;human ovarian surface epithelial cell lines :IOSEs	N/A	Down-regulation	RT-qPCR	N/A	So WK et al (76)
Acute myeloid leukemia	Mouse bone marrow tissue	N/A	N/A	N/A	Suppress leukemogenesis	Zhen Zhao et al (77)
Secondary acute myeloid leukemia	Human bone marrow tissue	N/A	Down-regulation	RT-qPCR;	N/A	Geiger, O (78)
**S**epsis-induced acute lung injury	SPRY4-MKO and SPRY4-MTG mice lung tissue; BALF; mBMDM, mAM, hPBMC;	LPS CPC STO	Up-regulation	WB RT-qPCR; GEO database	Exacerbates sepsis-induced ALI;facilitates sepsis-induced pulmonary inflammation and oxidative stress;	Rong Chen et al (1)
Glioblastoma multiforme	Glioblastoma-derived cell lines:DBTRG-05MG, U373, T98G, AM-38, BTL1529, BTL2177, BTL53, BTL1376 BTL2175, VBT72	Serum ; SPRY4 Overexpression cell model	Serum treatment: Up-regulation	WB	Inhibits proliferation and migration	Burcu Emine Celik-Selvi et al (79)
Glioblastoma multiforme	Human brain	N/A	Down-regulation	RT-qPCR	Suppress cell invasion; Associated with the favorable prognosis of GBM	Zhao B et al (80)
Breast cancer	Human breast cancer cell lines:MDA-MB-231	SPRY4 silence cell model	N/A	N/A	Increases sensitivity to Paclitaxel treatment; Suppress cancer stem cell properties	Hongyu Jing et al (36)
Lewis lung carcinoma	Spry4 KO mouse HEK-293T MEFs LLC B16F10	LLC and B16F10 cell-line-derived subcutaneous tumor model	N/A	N/A	Lack of SPRY4 promote tumor growth and angiogenesis.	Koji Taniguchi et al (27)
Gastric cancer	Human gastric tissue Gastric cell lines: SGC-7901 BGC-823	NA	Up-regulation	RT-qPCR TCGA database	Promote cell proliferation and migration; associated with poor prognosis	Pan Y et al (81)
Gastrointestinal stromal tumor	Human gastric tissue Human gist cell line: GIST882	Imatinib mesylate MEK inhibitor PI3K inhibitor	Treated with Imatinib : Down-regulation	RT-PCR cDNA Microarrays	Assess Imatinib therapy	Frolov A et al (82)
Gastrointestinal stromal tumor	Ba/F3, GIST-T1 Ecopack; Mouse with KIT/V558A mutation	SPRY4 depletion cell mode	N/A	N/A	Increase sensitivity to imatinib	Li, Shujing et al (83)
Hind limb and soft tissue ischemic injury	Spry4 KO mouse hind limb and soft tissue MEFs	Mouse hind limb and soft tissue ischemic model	N/A	N/A	Inhibit angiogenesis and reperfusion.	Taniguchi K et al (11)
Brain ischemic injury	Mouse brain;	Endothelin Brain injection of Spry4 siRNAs	N/A	N/A	Increase neuronal cell death and lesion area in subchronic phase	Klimaschewski L et al (7)
Experimental autoimmune encephalomyelitis	Spry4 KO mouse brain; Mouse naïve T cell SPRY4 Overexpression cell model	MOG CFA Calbiochem IL-1/6/23/TGF-β1	N/A	N/A	Exacerbate experimental autoimmune encephalomyelitis; positively regulates IL-1R1 expression	Fukaya T et al (6)
Endometrial adenocarcinoma	Human endometrium	N/A	Down -regulation	IHC	Involved in the pathogenesis of human endometrial adenocarcinoma	Zhang H et al (84)
Adenomyosis	Human eutopic endometria tissue	N/A	Down -regulation	IHC ISH	N/A	Guo Q et al (85)

MOG, Myelin oligodendrocyte glycoprotein; MEFs, Primary mouse embryonic fibroblasts; HEK-293T, human embryonic kidney (HEK) 293T cell; LLC cell, Lewis lung carcinoma; BMDMs, B16F10 melanoma cell; bone marrow-derived macrophages; PBMCs, Peripheral blood mononuclear cells; AMs, alveolar macrophages; BALF, Bronchoalveolar lavage fluid; (Spry4-MKO) mice, myeloid-specific Spry4 knockout; (Spry4-MTG) mice, macrophage-specific Spry4 transgenic; HUVEC, human vascular endothelial cells; WB, Western blotting; ICH, Immunohistochemistry; ISH, In situhybridization; IF, Immunofluorescence. NA, Not answered.

### 5.2 Glioblastoma

Glioblastoma (GBM) is the most common brain tumor with poor prognosis. In 2008, cancer genomics researchers suggested that the amplification and mutation of RTK genes are one of the main causes of glioblastoma (86), and the dysregulation of RTK-induced pathways is a key step in driving the carcinogenic potential of brain cancer. Zhao et al. found that SPRY4 mRNA is significantly lower in GBM compared to adjacent brain tissues, and that SPRY4 inhibits the malignant behavior of GBM tumors. Additionally, SPRY4 is an independent prognostic factor in GBM, with high expression of SPRY4 being associated with better prognosis. SPRY4 inhibits GBM invasion by inhibiting ERK phosphorylation and ETS-1-induced matrix metalloproteinase 9 (MMP9) expression (80). Matrix metalloproteinases (MMPs) can disrupt the tissue barrier of tumor invasion by promoting extracellular matrix degradation, facilitating tumor infiltration through the basement membrane and extracellular matrix. By exogenous expression of SPRY4, the proliferation and migration of GBM-derived cell lines can be suppressed, further confirming the potential role of SPRY4 in inhibiting GBM (79). Therefore, SPRY4 may serve as a potential therapeutic target, and its upregulation or restoration of function may provide therapeutic benefits for GBM patients. However, further research is needed to validate these findings and explore the clinical application of SPRY4-related therapeutic strategies.

### 5.3 Hepatocellular carcinoma and perihilar cholangiocarcinoma

Based on the anatomical location of the tumor, cholangiocarcinoma (CCA) can be divided into intrahepatic cholangiocarcinoma (ICC), perihilar cholangiocarcinoma (PHCC), distal cholangiocarcinoma (DCC), and other subtypes. PHCC is the most common subtype of CCA and has the poorest prognosis (87). Bo Qiu et al. confirmed that the expression of SPRY4 in PHCC tumor tissues was significantly lower than that in adjacent normal tissues, and overexpression of SPRY4 inhibited the malignant behavior of perihilar cholangiocarcinoma cells. The molecular mechanism by which SPRY4 exerts anti-cancer effects in perihilar cholangiocarcinoma is mainly related to ERK phosphorylation, which inhibits cell proliferation and migration. Additionally, SPRY4 is significantly associated with tumor size, lymphatic infiltration, and serves as an independent prognostic biomarker for PHCC (12). In conclusion, SPRY4 may act as a tumor suppressor in hilar cholangiocarcinoma by regulating ERK phosphorylation and affecting cell proliferation and migration, thereby impacting the malignancy of the tumor.

Currently, there are fewer studies on SPRY4 in hepatocellular carcinoma (HCC). Sirivatanauksorn et al. found that the expression of SPRY4 was decreased compared to normal liver tissues but did not investigate its role in HCC development (73). Qingxia Ma et al. found that in sorafenib-resistant HCC patients, the histone deacetylase HDAC4 in complex with the transcription factor MEF2D to form a complex that directly binds to the SPRY4 promoter region to inhibit the transcriptional level of SPRY4, leading to overactivation of the MAPK/ERK pathway. They further found that the HDAC4 inhibitor tasquinimod induced SPRY4 expression and inhibited ERK activity, eliminating the resistance of HCC cells to sorafenib treatment (65). These findings provide new insights into the treatment of HCC, especially in dealing with drug resistance.

### 5.4 Gastric cancer

In 2020, Chinese researchers analyzed the expression levels of SPRY4 in gastric cancer (GC) tissues from the TCGA database and found that SPRY4 was upregulated in human gastric cancer tissues, indicating that its expression levels were higher than in normal gastric tissues. Furthermore, the mRNA level of SPRY4 was validated in 78 cases of human gastric cancer tissues and non-gastric cancer tissues using RT-qPCR, confirming the high expression of SPRY4. High expression of SPRY4 is associated with several clinical pathological features of gastric cancer, including depth of infiltration, TNM staging, lymph node metastasis, and regional lymph node metastasis. High expression of SPRY4 is correlated with shorter median overall survival and median progression time, suggesting that it may serve as an adverse prognostic biomarker. In *in vitro* experiments, knockdown of SPRY4 expression in gastric cancer cell lines inhibited cell proliferation and migration. In *in vivo* experiments using a xenograft mouse model, the inhibition of tumor growth was observed upon knockdown of SPRY4 (81). Overall, this study reveals the potential of SPRY4 as an adverse prognostic biomarker and suggests its potential as a therapeutic target and prognostic biomarker. These findings provide important scientific evidence for future gastric cancer treatment strategies.

### 5.5 Breast cancer

Compared to normal human mammary epithelial cells (nHMEC), SPRY4 protein and mRNA expression were reduced in other breast cancer cell lines (BT20, MCF7, SKBR3, MDA-MB468, ZR-75), except for the MDA-MB231 cell line (36, 88, 89). Vanita Vanas et al. found that SPRY4 expression inhibits proliferation and migration of breast cancer cells by interfering with ERK phosphorylation and MAPK pathway activation (88). In contrast, inhibiting SPRY4 increased the protein level of β3-integrin, which promotes cell migration and invasion *in vitro* and lung metastasis *in vivo* in breast cancer cells (36, 90, 91). Hongyu Jing et al. first discovered that SPRY4 can regulate the characteristics of tumor stem cells. Knockdown of SPRY4 in MDA-MB-231 cells enhances tumor stem cell characteristics, including increased expression of CD133, CD44 subsets, and mammosphere formation. It also reduces sensitivity to paclitaxel treatment *in vitro* and enhances tumor formation in xenograft models, and this effect is not limited to MDA-MB-231 cells (36). In 2021, Ethan J. Brock et al. found that SPRY4 levels were significantly reduced in invasive ductal carcinoma compared to normal and ductal carcinoma *in situ* tissues. SPRY4 was highly expressed in ductal carcinoma *in situ* and decreased with the transition to invasive ductal carcinoma. They first revealed the role of SPRY4 in limiting the transition from pre-invasive lesions to invasive diseases, which was shown to be driven by a decrease in ERK/MAPK signaling transduction (56). MicroRNA-181 also has carcinogenic effects in breast cancer, partly due to targeting the 3’ untranslated region of SPRY4 (63). These findings suggest that SPRY4 may play a complex role in the occurrence, development, and treatment resistance of breast cancer. In some breast cancer cells, decreased expression of SPRY4 appears to be associated with the invasiveness and progression of cancer, while in other cases, the function of SPRY4 may be related to the inhibition of tumor growth and metastasis. Therefore, SPRY4 may have different biological significance and potential therapeutic target value in different subtypes and stages of breast cancer.

### 5.6 Lung cancer

In 2005, Winn et al. found that SPRY4 is highly expressed in non-small cell lung cancer cell lines that co-express Wnt-7a and Fzd-9 (92). When intracellular Wnt-7a binds to Fzd-9, it activates the MAPK signaling pathway. In this context, the upregulation of SPRY4 expression may serve as a feedback inhibitory response to this activation. Studies have also found that Wnt7A/Fzd9 signaling can increase Spry4 promoter activity through PPARγ, which further promotes the expression of SPRY4 (29). In 2006, they further discovered that the expression of Spry4 mRNA and protein was decreased in non‐small cell lung cancer (NSCLC) cell lines and poorly developed lung cell lines compared to untransformed human lung epithelial cell lines. In human NSCLC cell lines, SPRY4 inhibits cell proliferation, invasion and epithelial-mesenchymal transition. The MEK inhibitor trametinib inhibits the expression of SPRY4 in stromal-like KRAS mutant NSCLC, leading to the activation of AKT and ERK signals in stromal-like KRAS mutant lung cancer cells (29). This phenomenon explains why some patients with KRAS-mutant NSCLC may not respond well to MEK inhibitor therapy and highlights the need for combination treatment strategies to simultaneously inhibit MEK and other potential alternative proliferation signaling pathways, such as using inhibitors targeting the AKT signaling pathway. Additionally, osimertinib, a third-generation TKI targeting EGFR mutations, has been shown to decrease the expression of SPRY4 in PC-9 cells carrying EGFR mutations. This leads to the phosphorylation of AXL and sustained activation of the MAPK signaling pathway, which may be one of the reasons for the development of resistance (93). Recent studies have shown promising anticancer effects of SPRY4 in NSCLC. These effects are closely associated with the involvement of miR-411-5p/3p, which plays a key role in mediating the anti-tumor properties of Spry4 in this specific type of lung cancer. Research has demonstrated that the oncogenic microRNA-141 directly targets tumor suppressor genes such as SPRY4 and TXNIP, leading to their downregulation and promoting the progression of lung cancer (60). These research findings emphasize the complex role of SPRY4 in the development of NSCLC and how it interacts with tumor biology through different signaling pathways and molecular modulators. These discoveries provide potential targets for the development of new therapeutic strategies, including regulating the expression of microRNAs and combination therapy with inhibitors targeting specific signaling pathways.

### 5.7 Leukemia

Common chromosomal abnormalities in acute myeloid leukemia (AML) include complete loss or partial loss of chromosome 5 and/or 7 (94). These chromosomal losses may contribute to the occurrence and progression of leukemia. As mentioned earlier, human SPRY4 is located on the long arm of chromosome 5. So, what is the role of SPRY4 in AML? Gain-of-function mutations in the KRAS and NRAS genes lead to sustained activation of the RAS pathway, resulting in dysregulated proliferation and differentiation of bone marrow cells, which is associated with poor prognosis in AML (77). SPRY4, as a negative regulator of the RAS pathway, plays a role in inhibiting cancer development. Knockdown of SPRY4 accelerates the occurrence and progression of AML, mainly by increasing RAS signaling to promote cancer development (77). Furthermore, the expression levels of SPRY4 differ significantly among AML patients with different risk groups, with higher levels associated with the low-risk group (75). This suggests that the expression levels of SPRY4 may contribute to the prognostic assessment of high-risk patients. Further studies have confirmed the loss of SPRY4 in secondary AML, present in both early stages and during progression or relapse (78). Therefore, SPRY4 may play a tumor-suppressive role in AML. Further research is needed to explore how the loss of SPRY4 affects patient prognosis and how it may serve as a therapeutic target.

SPRY4 has been validated as a tumor suppressor gene in leukemia transgenic mouse models, and its disruption leads to the development of a lethal subtype in AML.

### 5.8 Testicular germ cell tumors

Testicular germ cell tumors (TGCTs) have a relatively low incidence rate in China, approximately 46,000 per 100,000, and are one of the most common malignancies in males aged 20-35. Through a genome-wide association study, Kanetsky et al. discovered that TGCTs have a genetic susceptibility. KITLG and SPRY4 are potential susceptibility genes (95). Variations in SPRY4 (rs4624820) are associated with a decreased risk of GCT (96). Further research has shown that SPRY4 gene variants may also play an important role in the susceptibility to pediatric and adolescent GCTs (97). In addition, a specific SNP (rs10463352) in SPRY4 demonstrates significant parent-of-origin effects, with a significantly higher risk when transmitted from the mother to the offspring than from the father (98). Das et al. further investigated SPRY4 and found that it is highly expressed (both at the mRNA and protein levels) in human TGCT samples, whereas it is expressed at a lower level in normal adult testes. In TGCT cell lines (833 K and NT2-D1), reducing SPRY4 expression through siRNA leads to decreased activation of the PI3K/Akt signaling pathway, resulting in reduced cell growth, migration, and invasion, thereby promoting tumor development (51). On the other hand, members of the miR-302 family act as oncogenes by inducing SPRY4 expression and activating the MAPK/ERK and PI3K/Akt signaling pathways (50). Overall, these findings contribute to a deeper understanding of the genetics of TGCTs and may provide information for the development of screening strategies and treatment methods for this disease.

### 5.9 Ovarian cancer

In China, epithelial ovarian cancer (EOC) ranks third in the incidence rate among female reproductive system tumors, with an increasing trend, but it has the highest mortality rate among female reproductive malignancies. Hua KT discovered that the histone methyltransferase G9a inhibits the expression of the tumor suppressor gene SPRY4, thereby promoting the proliferation and metastasis of ovarian cancer cells (99). This may be related to SPRY4’s inhibition of the Ras/MAPK pathway. Targeting histone methyltransferase could potentially become a new approach for therapeutic intervention. So WK found that the mRNA levels of SPRY4 showed no significant changes in samples from EOC patients of different subtypes, but the mRNA levels of SPRY4 were lower in human EOC cell lines (76). Similarly, other researchers found a significant decrease in SPRY4 protein in EOC patient tissues (74). Deletion of the SPRY4 gene is rare in high-grade serous carcinoma samples (76). This suggests that SPRY4 may not play a role in the progression of high-grade serous ovarian cancer. Similarly, although SPRY4 protein expression is decreased in EOC patient tissues, analysis revealed no significant correlation between SPRY4 expression and tumor stage, recurrence, post-treatment ascites, and survival time. So what is the function and regulatory mechanism of SPRY4 in human ovarian cancer? So WK found that knocking down SPRY4 inhibited AREG-induced cancer cell invasion and migration. However, the role of SPRY4 in prostate cancer and lung cancer is completely different (100). In different tumor microenvironments, the role of SPRY4 may vary, and such context-dependent functions increase the complexity of cancer treatment.

### 5.10 Gastrointestinal stromal tumors

Gastrointestinal stromal tumors (GISTs) are rare tumors, with an annual incidence rate of approximately 10 to 15 cases per million people worldwide (101). The K641E mutation in the receptor tyrosine kinase gene KIT has been found in both sporadic and familial cases of GIST in humans (102). Currently, targeted therapy with KIT inhibitors is the main treatment for GIST. Researchers have found that Spry4 may be a potential therapeutic target for GISTs with oncogenic KIT mutations in Kit(K641E) mouse models (103). Although the authors discovered the impact of GIST-associated KIT mutations on cell gene expression, they did not study it in depth. In 2003, researchers found that downregulation of SPRY4A is a reliable predictor of response to imatinib therapy in GIST (82). Further studies have found that the protein level of SPRY4 in extracellular vesicles can be used to evaluate the response to imatinib therapy and disease status before and after treatment (104). In 2015, Thys A found that knocking down SPRY4 promotes proliferation of icc cells in the gastric antrum and colon of mice, but no activation of the ERK pathway was detected (105). Further research confirmed that SPRY4 has an inhibitory effect in GIST, as it can bind to KIT and inhibit its expression and activity, thereby reducing cell survival and proliferation. Additionally, SPRY4 acts as a sensitizing factor for imatinib, enhancing the efficacy of the drug. However, the role of SPRY4 is invalidated due to secondary resistant KIT mutations that occur during the treatment of GIST (83). In conclusion, it is speculated that targeting SPRY4 and KIT in combination with inhibitors such as imatinib may be more effective in GIST treatment. The increased level of SPRY4 protein in extracellular vesicles may be related to the selection of GIST to avoid negative feedback interference in the KIT pathway.

## 6 SPRY4 and ischemic diseases

Barbara Haigl and colleagues have found that both hypoxic conditions and treatment with deferoxamine (DFO) can increase the expression of SPRY4 (106). The increased expression of SPRY4 may be achieved through enhanced gene transcription and mRNA stability. Koji Taniguchi and colleagues have discovered the mechanism of action of Spry4 under hypoxic conditions. Compared to wild-type (WT) mice, Spry4 knockout (KO) mice show greater resistance to hindlimb ischemia and soft tissue ischemia, as the absence of Spry4 accelerates neovascularization, resulting in significantly higher rates of hindlimb blood flow recovery in the KO mice after induction of hindlimb ischemia (11). These results suggest that SPRY4 may be a novel target for treating peripheral ischemic diseases. Additionally, studies have found that downregulation of the Spry2/4 genes has neuroprotective effects. This may be due to the promotion of astrocyte proliferation in the ischemic brain injury area by reducing Spry2/4 expression, resulting in reduced neuronal cell death and the size of the injury area (83). These findings further support the protective role of SPRY4 in limb ischemic injury and cerebral ischemic neural injury, providing potential directions for the development of new treatment methods or drug targets. However, further research is needed to validate these findings and evaluate the clinical feasibility of potential therapeutic strategies. (Refer to the **Table 2** for details).

## 7 Conclusion

SPRY4 protein assumes a pivotal role in the regulation of the RTK pathway, governing crucial aspects of organogenesis, developmental processes, and the emergence of malignant neoplasms. The significance of SPRY proteins varies across distinct cellular lineages, contingent upon the contextual milieu. In certain tumor types, the SPRY4 gene exerts its influence as a tumor suppressor, effectively quelling the malignant propensities of cancerous cells. Nevertheless, within the realm of gastric cancer, it metamorphoses into an oncogene, fueling the pernicious advancement of the ailment. Moreover, SPRY4 manifests its potential as a prognostic biomarker in specific cancers. The presence of oncogenic RAS mutations within certain tumors governs the dysregulation and functional manifestation of SPRY4. Furthermore, SPRY4 orchestrates the development of inflammatory maladies. At present, researchers ardently examine the expression and functionality of SPRY4 within tumor microenvironments, striving to fathom its intricate involvement in the malignant conduct of cancer cells. The relentless pursuit of utilizing SPRY4 as a promising target for anti-cancer therapeutics, aimed at enhancing tumor prognoses and surmounting drug resistance, remains an active field of investigation. However, the quest for small molecule activators that emulate the functionality of SPRY4 remains elusive.

## Author contributions

HP: Conceptualization, Writing – original draft, Writing – review & editing. RX: Software, Visualization, Writing – review & editing. YZ: Funding acquisition, Investigation, Supervision, Writing – original draft, Writing – review & editing.
